# Surgical treatment of type A Acute Aortic Dissection based on Geneva algorithm

**DOI:** 10.1186/1749-8090-10-S1-A313

**Published:** 2015-12-16

**Authors:** Burak Can Depboylu, Leon Finci, Patrick O Myers, Saziye Karaca, Dominique Vala, Jalal Jolou, Parmeseeven Mootoosamy, Marck Licker, Karim Bendjelid, Afksendiyos Kalangos, Mustafa Cikirikcioglu

**Affiliations:** 1 Division of Cardiovascular Surgery, Hospitals and Medical Faculty of Geneva, Geneva, Geneva, 1211, Switzerland; 2 Division of Anaesthesiology University, Hospitals and Medical Faculty of Geneva, Geneva, Geneva, 1211, Switzerland; 3 Division of Intensive Care, Hospitals and Medical Faculty of Geneva, Geneva, Geneva, 1211, Switzerland

## Background/Introduction

 Type A Acute Aortic Dissection (AAAD) is a highly deadly disease. Management of AAAD suspicion is extremely important in order to gain time and increase the likelihood of survival.

## Aims/Objectives

The aim of this study is to review our experience based on our local algorithm developed for the assessment and management of patients with AAAD suspicion over the last 8 years.

## Methods

All patients who underwent an emergency surgery for AAAD between 2007 and 2014 following our algorithm were assessed (Table). Their clinical situation at admission, evaluation, operative and postoperative data were evaluated retrospectively. Continuous variables were expressed as mean ± standard deviation; categorical variables were shown as frequency and percentage.

## Results

 A total of 68 patients were included during the study period. The mean age was 61 ± 13 years, with 42 men (65%). Supracoronary ascending aorta replacement was the primary surgical procedure (in 31 patients, 46%). The mean cardiopulmonary bypass, cross clamp and circulatory arrest times were 3.2 ± 1.6, 2.1 ± 1.2 and 0.4 ± 0.2 hours. Acute renal failure (27, 40%), re-operation (18, 26%) and pneumonia (14, 20%) were the main postoperative complications. Mean intensive care unit stay and hospitalization times were 5.8 ± 6.2 and 26.5 ± 53.5 days. There were 14 perioperative deaths (21%) and 18 hospital deaths (26%). The 6-year survival was 67.5%.

## Conclusion

Our institutional experience in managing AAAD is comparable to results from other centers. Standardizing management using an algorithm is important to gain time for rapid decision-making and having successful outcomes in centers with limited volume.

**Figure 1:**
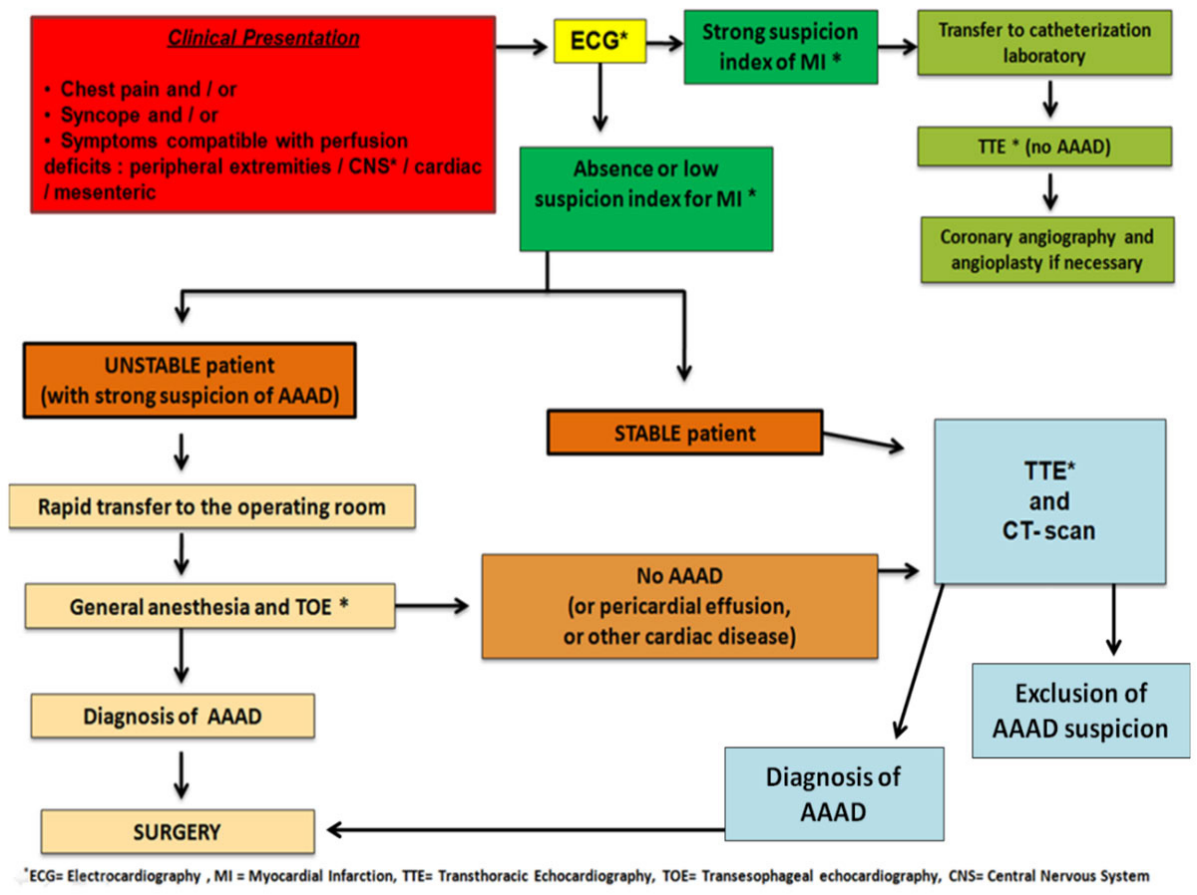
**Decision Algorithm: Suspicion on Acute Type A Aortic Dissection (AAAD)**.

